# Predictive models for estimating visceral fat: The contribution from anthropometric parameters

**DOI:** 10.1371/journal.pone.0178958

**Published:** 2017-07-24

**Authors:** Claudia Porto Sabino Pinho, Alcides da Silva Diniz, Ilma Kruze Grande de Arruda, Ana Paula Dornelas Leão Leite, Marina de Moraes Vasconcelos Petribú, Isa Galvão Rodrigues

**Affiliations:** 1 Pronto Socorro Universitário Cardiológico de Pernambuco–PROCAPE/UPE, Recife-PE, Brazil; 2 Universidade Federal de Pernambuco–UFPE, Recife-PE, Brazil; Temple University School of Medicine, UNITED STATES

## Abstract

**Background:**

Excessive adipose visceral tissue (AVT) represents an independent risk factor for cardiometabolic alterations. The search continues for a highly valid marker for estimating visceral adiposity that is a simple and low cost tool able to screen individuals who are highly at risk of being viscerally obese. The aim of this study was to develop a predictive model for estimating AVT volume using anthropometric parameters.

**Objective:**

Excessive adipose visceral tissue (AVT) represents an independent risk factor for cardiometabolic alterations. The search continues for a highly valid marker for estimating visceral adiposity that is a simple and low cost tool able to screen individuals who are highly at risk of being viscerally obese. The aim of this study was to develop a predictive model for estimating AVT volume using anthropometric parameters.

**Methods:**

A cross-sectional study involving overweight individuals whose AVT was evaluated (using computed tomography–CT), along with the following anthropometric parameters: body mass index (BMI), abdominal circumference (AC), waist-to-hip ratio (WHpR), waist-to-height ratio (WHtR), sagittal diameter (SD), conicity index (CI), neck circumference (NC), neck-to-thigh ratio (NTR), waist-to-thigh ratio (WTR), and body adiposity index (BAI).

**Results:**

109 individuals with an average age of 50.3±12.2 were evaluated. The predictive equation developed to estimate AVT in men was AVT = -1647.75 +2.43(AC) +594.74(WHpR) +883.40(CI) (R^2^ adjusted: 64.1%). For women, the model chosen was: AVT = -634.73 +1.49(Age) +8.34(SD) + 291.51(CI) + 6.92(NC) (R^2^ adjusted: 40.4%). The predictive ability of the equations developed in relation to AVT volume determined by CT was 66.9% and 46.2% for males and females, respectively (p<0.001).

**Conclusions:**

A quick and precise AVT estimate, especially for men, can be obtained using only AC, WHpR, and CI for men, and age, SD, CI, and NC for women. These equations can be used as a clinical and epidemiological tool for overweight individuals.

## Introduction

The distribution of anomalous body fat is recognized as an important predictor of cardiovascular risk [1,2]. Abdominal adipose tissue includes subcutaneous and visceral fat deposits that, when in excess, result in special risks to metabolic and hemodynamic parameters[3]. Robust evidence connects visceral obesity to a proatherogenic state[1–4], highlighting the importance of quantification of it for estimating metabolic risk and stratifying cardiovascular risk in patients in clinical practice.

The possibility of selectively measuring adipose visceral tissue (AVT) and subcutaneous tissue (AST) with due accuracy and reliability has been a notable contribution that has revolutionized the field of body composition1. Only imaging scans are able to quantify subcutaneous fat separately from visceral fat [5,6]. Thus, Computed Tomography (CT) represents the “gold-standard” for this type of evaluation [3,6]. However, its use is limited in clinical practice and in evaluating large population groups, due to the high cost and potential risk of exposure to radiation [7]. These limitations have resulted in only some clinical studies adopting this diagnostic exam for evaluating visceral obesitylevels, and consequently estimating the predictive value that this type of fat has in determining metabolic and cardiovascular alterations.

The search continues for a highly valid marker for estimating visceral adiposity that is a simple and low cost tool able to screen individuals who are at high risk of being viscerally obese. The usefulness of anthropometric indicators as “proxies” for indirectly estimating visceral fat depends on the degree to which these correlate with reference methods, which are those that provide a direst measure of AVT, since they allow it to be differentiated from subcutaneous abdominal fat [3]

The results regarding the superiority of one parameter in relation to others are still very controversial. While some results choose abdominal circumference (AC) as a better indirect indicator of intra-abdominal fat and cardiovascular risk, when compared to the body mass index (BMI) and waist-to-hip ratio (WHpR) [8–10], some indicate better performance for WHpR [11]. Moreover, other parameters suggested in the literature have not been effectively tested with regards to the predictive ability for AVT, such as waist-to-height ratio, neck circumference, conicity index, neck-to-thigh ratio, waist-to-thigh ratio, sagittal diameter, sagittal index, and body adiposity index.

Some authors have demonstrated the inappropriateness of anthropometric methods in estimating AVT when used in isolation. However, when these variables are included in a regression model, the precision of estimates can be optimized [2,12]. Thus, the aim of this study was to develop a predictive model for estimating visceral fat volume using anthropometric parameters that can be feasibly used in clinical practice.

## Research design and methods

A methodological study in which outpatients from a public hospital of reference in cardiology located in the Northeast of Brazil were recruited. At this outpatient clinic, the public seen is predominantly composed of individuals with non-infectious chronic illnesses, including systemic arterial hypertension, diabetes mellitus, metabolic syndrome, and dyslipidemia.

The sample was constructed based on voluntary adhesion, using overweight individuals of both sexes and aged ≥20. Excluded were individuals with hepatitis and/or splenomegaly, ascites, recent abdominal surgery, pregnant women, and women that had had children up to 6 months before screening, all characteristics that can influence intra-abdominal and/or anthropometric measures. Also considered ineligible were individuals with physical limitations (the amputation of some limb) that made obtaining anthropometric measures impossible. Excess weight was established based on a BMI≥25kg/m^2^ for adults and a BMI≥27kg/m^2^ for seniors [13].

Considering an error α of 5%, an error β of 20%, with an estimated average correlation between anthropometric variables and AVT of 0.5 (p) and a variability of 0.15 (d2), and using the formula n = [(Zα/2 + Z β/2)2 x (p x (1—p)] / d^2^, a minimum sample size of 88 individuals was obtained. In order to correct for potential losses, 20% was added to the sample, resulting in 110 sample units.

Adipose visceral and subcutaneous tissues were evaluated using Computed Tomography (CT), using a Philips Brilliance CT-10 slice tomography (*VMI Indústria e Comércio Ltda*, Lagoa Santa, MG, Brazil). The exam was carried out by a single observer (a medical radiologist) with the patients completely fasted for four hours. The tomographic cross-section was obtained with radiographic parameters of 140 kV and 45 mA, at the lumbar vertebra level L4, with a thickness of 10 mm. The total area of abdominal fat and the visceral fat area were outlined manually with a free cursor contouring each region. The entire skin surface was excluded from the marked area. AVT area was determined taking the internal borders of the abdominal rectus, internal oblique, and lumbar quadrate muscles as limits, excluding the spine, and including retroperitoneal, mesenteric, and omental fat. All of the fatty areas were described in cm2. In order to identify adipose tissue, the density values-50 and -250Hounsfield units were used [14,15].

The following anthropometric parameters were evaluated: BMI, AC, WHpR, waist-to-height ratio (WHtR), sagittal diameter (SD), sagittal index (SI), conicity index (CI), neck circumference (NC), neck-to-thigh ratio (NTR), waist-to-thigh ratio (WTR), and body adiposity index (BAI).

Weight and height were measured according to techniques prescribed by Lohman, Roche, and Martorell [16], using electronic scales (Welmy®, Santa Bárbara d’Oeste, SP, Brazil), with a 150Kg capacity, 100g division, and a stadiometer attached, with 1 mm precision. AC was calculated with an inelastic metric tape, with 0.1 cm precision, directly over the skin at the mid-point between the last rib and the iliac crest. The bone markings of the last rib and iliac crest were located and palpated by the examiner at the level of the midaxilary line. The measuring tape was placed in a horizontal line around the abdomen in the location mentioned above and special attention was paid to guarantee that the tape was parallel to the floor [17].

Hip circumference was obtained by measuring the hip region at the area of greatest protuberance [18]. NC was measured with an inelastic metric tape with the individuals standing up erect with their heads positioned in the Frankfurt horizontal plane and looking forward. The metric tape was placed perpendicularly over the neck axis at the mid-point of the cervical spine to the mid anterior of the neck. In men with laryngeal prominence, the NC was measured below the prominence [19].

The SD measurement was carried out with the individuals in a supine position, using an anthropometer to measure the distance between the dorsum in contact with the surface and the highest point of the abdomen, between the last rib and the iliac crest [20]. The thigh measurement was obtained on the right side of the body, at the mid-point between the inguinal fold and the proximal edge of the patella [3].

The BMI was obtained from the equation: Weight(kg)/Height(m)^2^ and the WHpR was determined by the abdomen (cm) and hip (cm) parameter ratio. The WHtR was evaluated using the ratio between abdominal circumference (cm) and height (cm). For the CI calculation the waist circumference and height measurements, expressed in meters, and body weight (kg) were considered, in accordance with the following mathematical equation [21]: Waist circumference (m)/ {0.109 x √[(Body weight (kg)/Height (m))]}.

The NTR was determined by the ratio between neck circumference (cm) and thigh circumference (cm). The WTR was obtained using the ratio between waist circumference (cm) and thigh circumference (cm) [3,22]. The SI was obtained using the ratio between sagittal diameter and thigh circumference: SD (cm)/Thigh circumference (cm) [23]. The BAI was obtained using the equation: [Thigh circumference (cm) / Height (m)^1,5^]– 18.

For each anthropometric point evaluated, a double measure was obtained by a trained examiner. When the difference calculated between the measures was greater than 0.1 cm or 0.1kg, a third measurement was carried out. The final measurement considered was the average between the two closest values.

The study protocol was guided by the ethical standards for research involving human beings, set out in National Health Council resolution 466/12, and was submitted for evaluation by the University of Pernambuco (*UPE*) Committee on Ethics and Research with Human Beings, and approved under protocol number 271.400/2013. The individuals were previously informed of the research objectives, as well as the methods adopted, and with their agreement, they signed an informed consent form.

The data were analyzed with the help of the Statistical Package for Social Sciences–SPSS program, version 13.0 (SPSS Inc., Chicago, IL, USA). The continuous variables were tested with regards to distribution normality using the Kolmogorov Smirnov test, and as they presented a normal distribution they were described in average and standard deviation form. For the description of proportions, an approximation of the binomial distribution to the normal distribution was carried out using a confidence interval of 95%. The Student t test for independent samples was used to compare between averages of the anthropometric parameters and visceral fat between sexes. The proportions were compared using the Pearson Chi Squared test.

In the multivariate analysis a stepwise multiple linear regression was used for age and anthropometric variables as independent variables (or predictors) and AVT was used as a response variable. A backward regression analysis was adopted for the model and the Wald test was used to verify the statistical significance of the model.

The anthropometric parameters that presented a connection with AVT in the univariate analysis were included in the multiple regression and the models in which the variables presented a VIF (variance inflation factor)<10 [24] were considered. The variables with superior VIF were taken from the regression and a new model was constructed without them. Simple linear regression was used to evaluate the explanatory power of the predictive equation for AVT in relation to AVT volume determined by CT. Statistical significance was established when the p value<0.05.

## Results

110 patients were recruited, and after eliminating one loss, 109 individuals composed the final study sample. The average age was 50.3(±12.2), varying from 20 to 75. There was a predominance of females (74.3%; CI_95%_:65.0–82.2) and the BMI varied from 25kg/m^2^ to 45kg/m^2^. No statistically significant difference was verified with relation to age distribution, and prevalence of DM and SAH between sexes. The men presented greater absolute and relative AVT (p<0.001), when compared to the women (Table 1). The sample's racial composition was 38.5% white, 10.1% black and 51.4% brown.

**Table 1:** Comparability of the characteristics of the patients included in the study in accordance with sex.

Variables	Men (n = 28)	Women (n = 81)	p-value*
Age, year (mean/SD)	49.9 (±13.7)	50.5 (±11.8)	0.817*
Arterial hypertension (%, CI_95%_)	67,9 (47,6–84,1)	59,3 (47,8–70,0)	0.420^§^
Diabetes *Mellitus* (%, CI_95%_)	25,0 (10,7–44,9)	21,0 (12,7–31,5)	0.659^§^
AVT (cm^2^)	378.9 (±118.7)	258.6 (±75.4)	<0.001*
AST (cm^2^)	506.3 (±162.2)	540.9 (±145.6)	0.294*
%AVT (mean/SD)	43.2 (±10.3)	32.7 (±8.4)	<0.001*
%AST (mean/SD)	57.1 (±10.2)	66.9 (±8.4)	<0.001*

*Student t test for unpaired data

^§^ Chi Squared test.

SD: Standard Deviation; CI_95%:_95%Confidence Index; BMI: Body Mass Index; AVT: Adipose Visceral Tissue; AST: Adipose Subcutaneous Tissue. %AVT: Proportion of visceral fat in relation to total abdominal fat concentration. %AST: Proportion of subcutaneous fat in relation to total abdominal fat concentration.

Higher averages for the anthropometric parameters that reflect body fat distribution were observed in the males (AC, WHpR, SD, CI, NC, WTR, NTR), when compared to the women. However, when the BAI was evaluated, which reflects the percentage of body fat via a mathematical model that uses hip circumference and height measures, a higher value was verified among the women (p<0.001) (Table 2).

**Table 2:** Distribution of anthropometric parameters (Mean/Standard Deviation) in accordance with sex.

Anthropometric parameters	Men (n = 28)	Women (n = 81)	p-value*
BMI, kg/m^2^	33.1 (±4.9)	33.5 (±5.3)	0.715*
AC (cm)	112.9(±12.5)	103.2(±11.0)	<0.001
WHtR	0.7(±0.1)	0.6(±0.1)	0.503
WHpR	1.0(±0.1)	0.9(±0.1)	<0.001
CI	1.3(±0.1)	1.2(±0.1)	<0.001
SD (cm)	29.3 (3.2)	24.7 (±3.0)	<0.001
SI	0.5 (±0.1)	0.4 (0±0.1)	<0.001
NC (cm)	42.3(±3.1)	36.9 (±2.9)	<0.001
NTR	0.8(±1.4)	0.6(±0.1)	<0.001
WTR	2.1 (±0.2)	1.7 (±0.3)	<0.001
BAI	32.4 (±4.2)	39.8 (±6.3)	<0.001

*Student t test for unpaired data.

BMI: Body Mass Index; AC: Abdominal Circumference; WHtR: Waist-to-Height Ratio; WHpR: Waist-to-hip ratio; CI: Conicity Index; SD: Sagittal Diameter; SI: Sagittal Index; NC: Neck Circumference; NTR: Neck-to-Thigh Ratio; WTR: Waist-to-Thigh Ratio; BAI: Body Adiposity Index.

In the multiple regression analysis, five models were presented for the males and four for the females. The model that included the AC, WHpR, and CI variables was considered the best predictive model for AVT in men, as shown in equation 5: AVT = -1647.75+ 2.43 (AC) + 594.74 (WHpR) + 883.40 (CI), with an adjusted regression coefficient (R^2^) of 64.1%. The inclusion of other variables in the model (equation from 1 to 4) did not increase explanatory ability (Table 3).

**Table 3:** Multiple linear regression coefficients of predicative equations (eq.) for estimating adipose visceral tissue (AVT) in overweight individuals.

**Men (n = 28)**													
**Eq.**	**Constant**	**Standard Error**	**Age**		**AC**	**WHtR**		**WHpR**	**CI**	**NC**	**WTR**	**R**^**2**^ **(%)**	**R**^**2**^**(%) Adjust.**
**1**	-1440.50	371.62	1.89		6.71	-778.57		662.62	626.78	-0.70	13.62	72.4	62.7
**2**	-1451.62	351.73	1.90		6.61	-776.33		659.04	620.90	-	14.67	72.3	64.4
**3**	-1473.13	320.07	1.90		6.52	-757.28		677.83	644.01	-	-	72.3	66.0
**4**	-1666.84	295.61	-		4.68	-534.60		794.42	821.70	-	-	69.8	64.5
**5**	-1647.00	296.69	-		2.43	-		594.74	883.40	-	-	68.1	64.1
**Women (n = 81)**													
**Eq.**	**Constant**	**Standard Error**	**Age**		**BMI**	**WHtR**		**WHpR**	**SD**	**CI**	**NC**	**R**^**2**^ **(%)**	**R**^**2**^**(%) Adjust.**
**1**	-684.75	135.81	1.53		2.70	-336.13		200.16	10.04	287.56	5.82	45.3	40.1
**2**	-664.75	132.58	1.42		-	-158.68		125.40	10.46	258.21	7.25	44.9	40.5
**3**	-658.01	132.41	1.54		-	-136.99		-	10.47	325.94	7.33	44.2	40.5
**4**	-634.73	130.56	1.49		-	-		-	8.34	291.51	6.92	43.4	40.4

BMI: Body Mass Index; AC: Abdominal Circumference; WHtR: Waist-to-Height Ratio; WHpR: Waist-to-hip ratio; CI: Conicity Index; SD: Sagittal Diameter; NC: Neck Circumference; WTR: Waist-to-Thigh Ratio.

For the women, the model chosen to predict AVT (equation 4) involved the variables: age, SD, CI, and NC: AVT = - 634.73 + 1.49 (Age) + 8.34 (SD) + 291.51 (CI) + 6.92 (NC), with an adjusted regression coefficient (R^2^) of 40.4%. The addition of other variables did not cause any increase in the model’s explanatory power (Table 3).

A VIF <10 was defined as a criterion for model selection, indicating that there was no collinearity bias. The VIF of the variables included in the regression model for males varied from 1.31 to 1.76, while in females it was 1.11 to 1.32.

**Fig 1:**
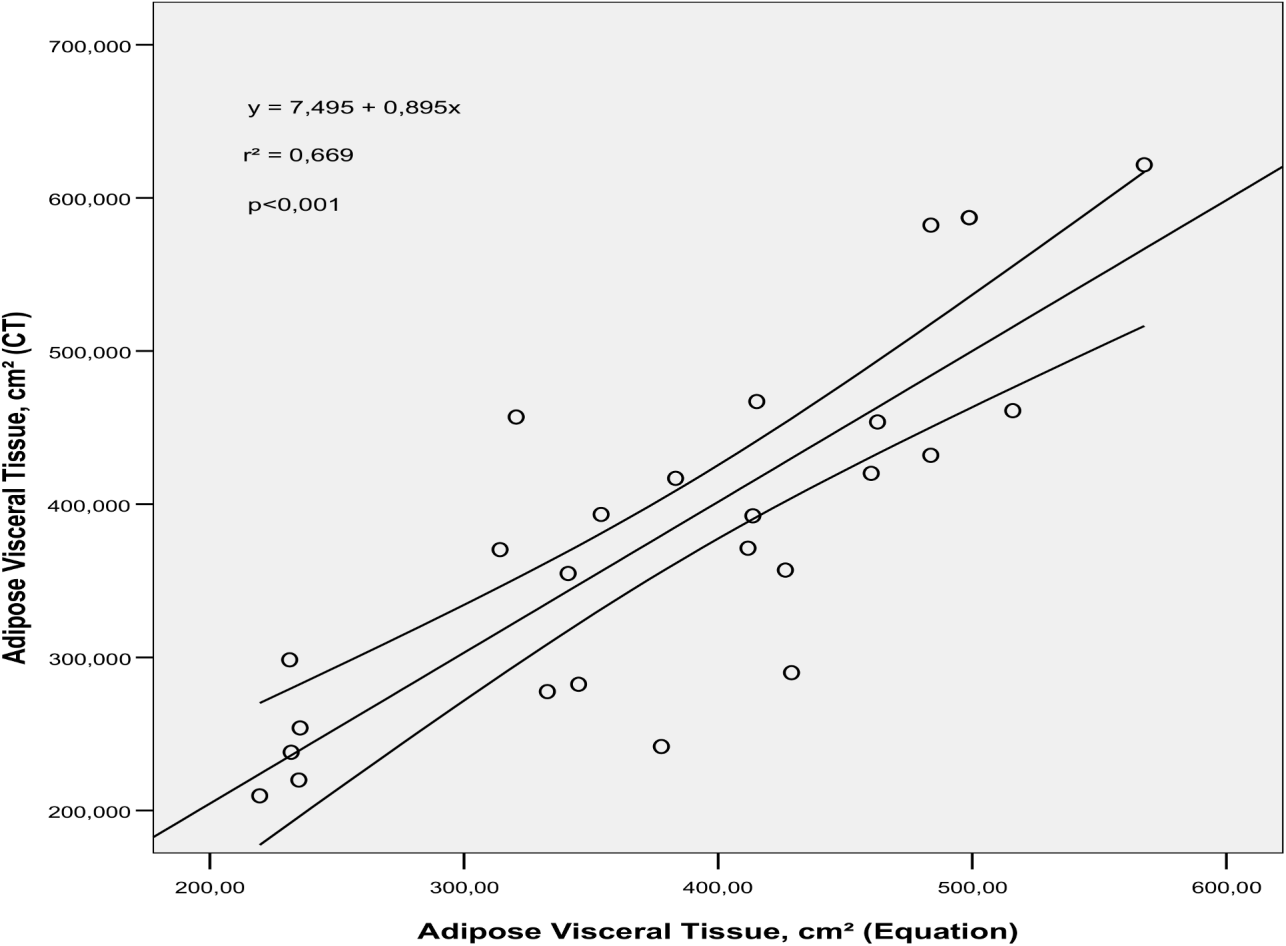
Simple linear regression between adipose visceral tissue (AVT) volume determined by the predictive equation and AVT volume obtained using computed tomography (CT) in adult overweight men (p<0.001).

**Fig 2:**
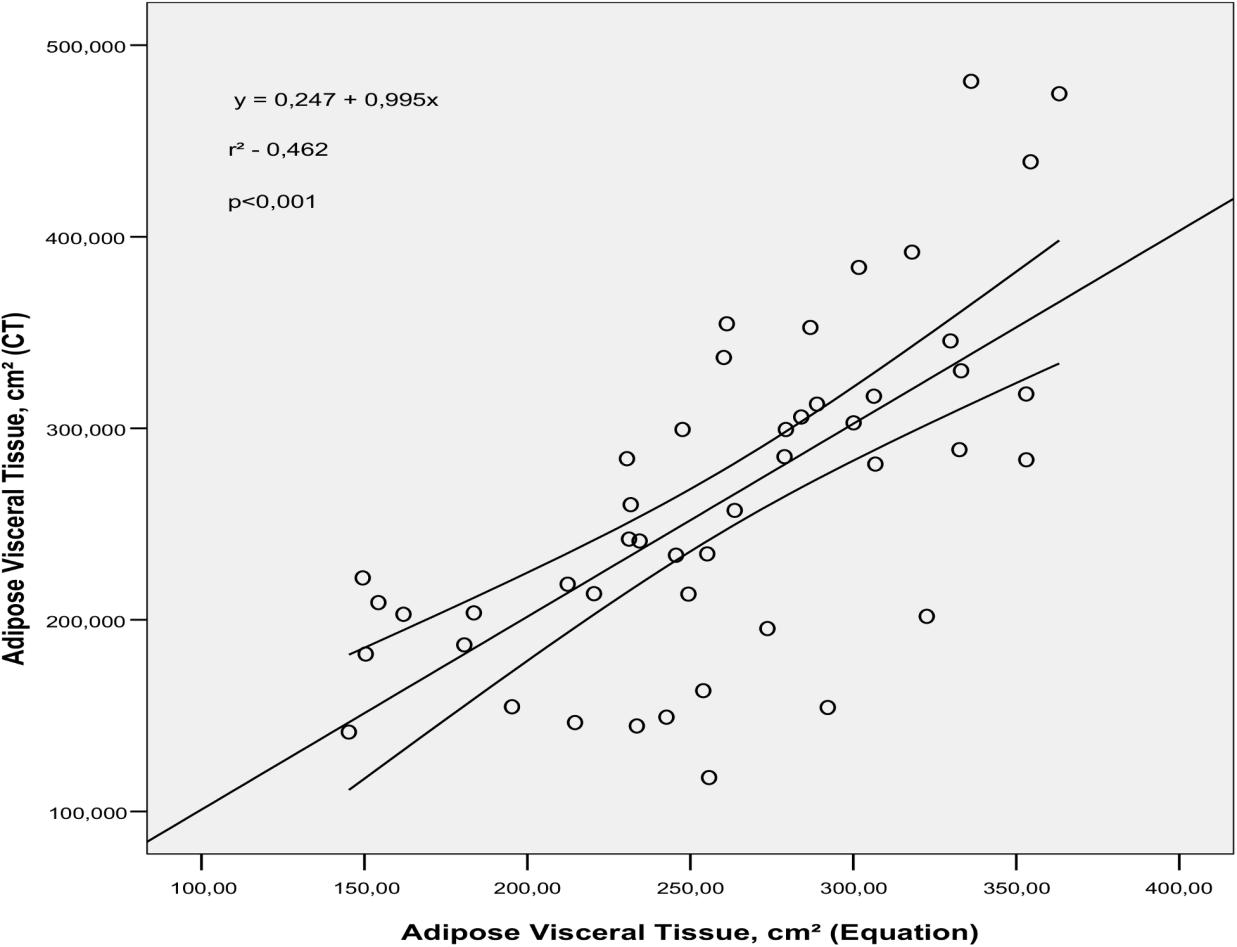
Simple linear regression between adipose visceral tissue (AVT) volume determined by the predictive equation and AVT volume obtained using computed tomography (CT) in adult overweight women (p<0.001).

The predictive ability of the equations developed was 66.9% for males and 46.2% for females in relation to AVT volume determined by CT (p<0.001), as can be observed in Figs 1 and 2, respectively.

## Discussion

In this study simple equations were developed for predicting AVT based on anthropometric measures and indices that are easy to obtain and can be feasibly reproduced in clinical practice and in evaluating large population groups. These equations can be used to estimate AVT area in overweight individuals of both sexes aged between 20 and 75. Considering that AVT constitutes an independent risk factor for cardiometabolic alterations, estimating this abdominal adipose tissue sub-compartment represents an important tool for screening individuals at risk of being viscerally obese.

There are not a large number of equations for predicting AVT area available in the literature. Moreover, the results from studies cannot be rigorously compared, given the different characteristics of the populations investigated. Therefore, generalization of the applicability of a predictive model for estimating bodily compartments should be made with great caution, observing the age, sex, adiposity level, and racial characteristics of the population in which it has been validated.

The greater AVT concentration in men, for the same BMI, age, and subcutaneous adiposity level, reveals a greater predisposition in men for accumulating fat viscerally, with this result being consistent with previous investigations [20,25,26]. Thus, considering the notable differences in the distribution pattern of body fat and in AVT accumulation, the need for different predictive equations to be developed for the sexes is evident, or at least for sex to be inserted as a variable into the model.

The predictive equation developed for males presented a higher prediction level (64.1%), compared to the regression model obtained for females (40.4%), and was relatively similar to previously published results [12,27,28]. Goel et al [12], in evaluating 171 Asians with an average age of 32.2 and an average BMI of 22.9km/m2, developed an equation with a predictive ability of 52.9%. Brundavani et al [27] described a model with 74% prediction in men aged from 40 to 79.

It is important to consider that although the equation proposed for women was only able to explain 40% of AVT variability, since it is impossible to evaluate visceral fat using imaging methods, applying an equation could be an alternative strategy for having a screening tool for individuals at risk of being viscerally obese.

Statistically, the best predictive equations for AVT in our study involved three variables for men and four for women. The number of variables inserted into a regression model represents an important aspect to be considered when selecting a predictive equation, considering that with each variable added to the model a potential source of error is inserted into the estimate, limiting its applicability in practice. Thus, we recommend the models with the smallest number of variables involved. Adding more variables would make the model more complex without adding any significant increase to the estimate. Other authors have also reported that the inclusion of more than three predictive variables for estimating AVT increased the standard deviation and did not result in an improvement in the model’s explanatory power [2,29].

The final predictive model for males included AC, WHpR, and CI. The CI incorporates three important measures: weight, height, and AC, the latter being common to the other parameters. It was demonstrated that this index can be quite sensitive in detecting visceral obesity, especially in men, and can detect alterations in fat distribution, allowing for comparisons between individuals that have different body measures of fat and height [5]. WHpR, in turn, has also been listed as an important predictor of AVT. However, these findings are controversial, with it being observed in some results that this parameter presented a strong correlation with AVT [11] and it being described in others that this indicator can represent subcutaneous fat much more than visceral fat [5].

Some authors have indicated an increased correlation between AC and WHpR, and so the two predictors are rarely used in the same estimation model, in order to avoid collinearity problems, which would affect the regression estimate. In our study, in the predictive model for men, the two variables were included, but the VIF of the equations selected for both sexes in our study was lower than 2.0, justifying maintaining all of them. VIF> 10 increases the possibility of collinearity among predictor variables and may decrease the regression model’s confidence, which was not observed in our results.

Age, SD, CI, and NC were the parameters inserted into the predictive equation developed for women in this investigation. Age is a very important variable for evaluating body composition, considering the physiological modifications that accompany the ageing process, in which a reduction in fat free mass and an increase in total fat mass are observed, with a notable increase in fat stored in the intra-abdominal and intra-muscular anatomical sites, instead of in the subcutaneous region, as generally occurs in young adults [30]. Therefore, the inclusion of age can indicate that the model is able to predict AVT variations that can occur *paripassu* with age progression. The insertion of age into the female model reproduces some of the previous results that have aimed to estimate AVT [31,32], with age appearing, in fact, to interfere in determining AVT.

Some evidence indicates that SD isthe anthropometric parameter with the greatest power to explain AVT variability [5,20,32]. SD represents abdominal height, constituting a simple measure with good reproducibility and accuracy, based on the fact that in individuals in a position of dorsal decubitus, visceral fat accumulation maintains abdominal height in the sagittal sense, at the same time that subcutaneous fat is reduced, because it spreads to the sides, due to the force of gravity [20,33].

The relationship between NC and AVT has not yet been extensively evaluated. One investigation carried out by Yang et al [34] indicated that NC was a powerful marker of visceral fat quantity diagnosed by CT. This possible relationship has been attributed to the fact that systemic free fatty acids are mainly determined by fat in the upper part of the body, it thus being suggested that fat deposited in the neck region could play an important role in the pathogenesis of cardiovascular risk factors, especially in obese individuals [35].

The anthropometric parameters inserted into the previously validated predictive models are varied and seem to depend on the characteristics of the population for which they were validated. Nagai et al.[36] developed and validated an equation to predict AVT in men with an average age of 44.4 ±18.4 using WHtR and triglyceride serum level as variables (AVT = 857.66 x WHtR + 0.22 x TG– 378.31), presenting high sensitivity and specificity (0.833 and 0.900, respectively). Other equations that have found precise results in AVT estimates were proposed by Ran et al [37], in which the variables AC and age were used for males, and WHpR, weight, and age for females, and by Liu et al [38], who developed a model containing BMI and AC to estimate visceral area in male type 2 diabetic patients.

When the equations were applied in this study sample and the values compared with the reference model (CT), we verified good explanatory power in the predictive model in estimating AVT (r^2^ = 66.9% for males and r^2^ = 46.2% for females). However, it is worth noting that cross validation would be important for confirming these findings.

Some limitations should be considered when interpreting of the data presented. One of these aspects is the fact that the participants in the study had a high level of adiposity, and therefore application of the equation for individuals with different adiposity levels is limited. The possibility of having an equation available that can be applied to estimate AVT in overweight individuals is particularly important in the follow up for these individuals in clinical practice and as a monitoring tool during therapeutic interventions.

The main inconvenience in using predictive equations relates to the fact that they are validated in specific groups, therefore limiting their use in different populations, ethnicities, age groups, and adiposity levels. It is important for these equations to be validated for future use as AVT predictors and their applicability compared with preexisting equations.

Another aspect that should be considered is that the Brazilian population has specific racial characteristics, marked by great miscegenation between black and white races, and caution should be used in employing the equation for populations of other ethnicities. Thus, generalized use of the equation for populations of other races should be preceded by validation in different groups.

## Conclusions

This study showed that a quick and precise AVT estimate, especially for men, can be obtained using only AC, WHpR, and CI for men, and age, SD, CI, and NC for women, These equations can be used as a clinical and epidemiological evaluation tool for overweight individuals, allowing AVT volume to be quantified based on anthropometric measures.

Validation of the predictive models developed in this study is recommended in other population groups so that the possibility of their use can be broadened.

## Supporting information

S1 Database (SAV)
